# Organizational adaptation to working from home in a crisis situation (COVID-19): the interaction between leaders’ openness and followers’ voice

**DOI:** 10.3389/fpsyg.2023.1181807

**Published:** 2023-05-25

**Authors:** Barnabás Buzás, Klára Faragó

**Affiliations:** ^1^Doctoral School of Psychology, Eötvös Loránd University, Budapest, Hungary; ^2^Institute of Psychology, Faculty of Education and Psychology, Eötvös Loránd University, Budapest, Hungary; ^3^Department of Organisational and Leadership Psychology, ELTE Faculty of Education and Psychology, Budapest, Hungary

**Keywords:** adaptive leadership theory, leadership openness, employee voice behavior, work from home, COVID-19, psychological safety, intrinsic work motivation, affective organizational commitment

## Abstract

**Introduction:**

We investigated the effect of time spent at home on employee voice behavior and leadership openness during Covid 19. According to DeRue’s adaptive leadership theory which offers an interactionist perspective to explain adaptive organizational behavior during an environmental crisis, we proposed that in the WFH’s (work from home) reduced and limited communication space, leaders, who need more feedback, will encourage employees to express their opinions and will show more willingness to listen to them. Meanwhile, employees will ask more questions and make more suggestions to alleviate uncertainty and misunderstanding.

**Methods:**

Using an online questionnaire, a cross-sectional study (*N* = 424) has been carried out with employees working from home for a different amount of their working time during the pandemic. Data were analyzed using structural equation models (SEM) in which the effect of leadership openness on employee voice behavior was assessed through the mediation of affective commitment, psychological safety, and intrinsic motivation.

**Results:**

The results showed that in the WFH situation, time spent in home office had a low but significant direct negative effect on promotive voice behavior. At the same time, leadership openness was growing with the amount of time spent at home. Leadership openness counteracted the negative effect of WFH on voice behavior: although leadership openness did not have a direct significant effect on voice behavior, it had a positive effect on psychological safety and work motivation which, in turn, influenced positively both promotive and prohibitive voice behavior. Employee’s voice, for its part, further augmented leadership openness.

**Discussion:**

In our research we could demonstrate the contingent nature and the mutual influence patterns and feedback loops of leaders-employees exchange. In the WFH situation the openness of the leader is growing with the amount of time spent at home and with the amount of promotive voice manifested by the employee. In consent with DeRue social interactionist adaptive leadership theory, a mutually reinforcing process of leadership openness and employee voice could be demonstrated. We argue that leadership openness is a key factor to motivate employee voice behavior during WFH.

## Introduction

On the 11th of March, 2020, the World Health Organization (WHO) stated the pandemic of COVID-19 (WHO, 2020). Then, the COVID-19 virus spread rapidly worldwide and threatened the health of citizens. Many countries applied the tactic of “social distancing” in order to avoid a global health crisis. However, this tactic greatly influenced the life of society. Social distancing is not just affected the daily life of citizens but has caused economic consequences (Kraus et al., 2020). From an organizational point of view, the COVID-19 pandemic and the related governmental actions enhanced the effects of the so-called VUCA world and led to hyper-fast changes and uncertainty. Many companies introduced WFH in order to help “social distancing.” In the Western economies, 30 to 50 percent of people have worked remotely during the pandemic (Kohont and Ignjatović, 2022). According to KSH (Hungarian Central Statistical Office) (2021), the ratio of employees who regularly or occasionally work from home (WFH) emerged from 2.3% (February 2020) to 12.9% (April 2021, the time of data collection) in Hungary.

The prolonged and mandatory WFH posed a challenge to leaders worldwide. Due to the pandemic and the WFH, communication between leaders and followers changed radically (Marino and Capone, 2021). Leaders cannot supervise closely their subordinates, and employees do not get quick feedback from their supervisors. Online communication diminishes the possibility of exchanging nonverbal messages and reading metacommunicative signs. Communication is essential for smooth organizational functioning. The objective of our study is to investigate the dynamic and mutual process of goal-directed communication focusing on employees voice and leaders openness.

How can organizations cope with the challenge? Several authors summarize what leaders should do to promote the organization’s goal to maintain their leadership and to help employees to surmount the difficulties they encounter in the new WFH circumstances. Virtual leaders must invest more time and effort in the human side of leading because they need to deal with followers’ isolation, sustain team cohesion and shape the new norms of collaboration and communication (Contreras et al., 2020; D’Auria and De Smet, 2020; Stoker et al., 2022). Their main challenges are to clarify roles, define effective communication methods, demonstrate empathy, build trust with the staff, and provide emotional support (Levasseur, 2012; Slade, 2015; Wittmer and Hopkins, 2022). The above views consider the leader-subordinate relationship as a one-sided process, where a good answer to the crisis depends on the leader’s quick and smart reaction, and which is followed by the employees’ adequate reaction. This view about leaders, as heroes saving the world, does not take into consideration that in a crisis situation, leaders need help, feedback, and meaning-making from the part of the followers as well.

Leaders and followers need to solve the problems caused by the reduction of communication possibilities together. To uncover the mechanism of this mutual problem-solving process we turn to DeRue’s (2011) adaptive leadership theory. In our view, this theory is especially suitable to discern the adaptation process to the radically changing environment by analyzing the interdependent and interactive nature of leaders-followers reactions to the WFH. DeRue claims that theories of leadership usually disregard the social and contextual embeddedness of leadership in an organization, and present leadership as an individualistic, hierarchical, one-directional process.

In the leader-follower relationship, a reciprocal influence pattern emerges and evolves, enabling the development of the leading–following relationship over time (DeRue, 2011). This reciprocal influence pattern can change the direction of influence, altering the paths allotted by the formal structure, and encouraging subordinates to engage in actions that are traditionally viewed as leader behaviors (such as employee voice), and leaders to act as followers (such as listening to the voice). Exceeding the one-sided influence view by a double-interact perspective makes it possible to take into consideration the contingent nature of response patterns, where mutual influence patterns and feedback loops (future action is a function of prior interactions) are apprehended. DeRue’s theory changes the current focus from decontextualized, individualistic, hierarchical, one-directional concept of leadership and concentrates on its mutual, interactional nature and dynamics.

In our study, we try to grasp the mutual adaptation process to the changed communication situation through employee voice and leader openness. Employee voice, as a manifestation of OCB (Podsakoff et al., 2000; LePine et al., 2002; Dyne et al., 2003) helps to find a solution to new challenges in an organization (Walz and Niehoff, 2000; Detert et al., 2013). Employees’ ideas and concerns are particularly helpful for organizations during the COVID-19 pandemic because it enables the communication and interpretation of new work routines, and the exchange of views about the different practice suggested by the leaders or invented by the employees. According to De Rue, employees who share their ideas about work processes, practices, communication, and cooperation, and who cultivate voice behavior are practicing a sort of leadership function.

This approach is innovative from various perspectives. In our knowledge there is no empirical study to grasp the mutual adaptation process to the changed goal directed communication situation through employee voice and leader openness. Therefore, we investigated whether WFH has an impact on leader openness and whether leader openness influences voice behavior and vice versa, whether employee’s voice influences leaders openness. Furthermore, we included in our study three mediating variables that are predictors of voice behavior: psychological safety (Liang et al., 2012), intrinsic motivation (Uğurlu and Ayas, 2016; Jaaffar and Samy, 2023), and affective commitment (Nisar et al., 2020; Cheng et al., 2022).

### Literature background

Most authors agree about the increasing importance of voice during the pandemic, and especially in the new WFH situation because voice supports the adaptability and the well-being of organizations (Kim and Lim, 2020; Ta’Amnha et al., 2021; Prouska et al., 2022). On the same time there are serious impediments to speak up, e.g., stress (NG and Feldman, 2012), social empathy silence (Prouska and Psychogios, 2018; Fuchs and Reichel, 2021), and digital communication difficulties (Mao and DeAndrea, 2019).

Organizational voice represents the willful expression of people’s views to influence organizational activities in a constructive way to enhance productivity and development (Van Dyne and LePine, 1998; Bowen and Blackmon, 2003). Liang et al. (2012) distinguish two factors of employee voice behavior: promotive voice (expression of ideas and constructive suggestions in order to improve efficiency) and the prohibitive voice (expression of concerns about potential threats, malicious organizational behaviors, and the low performance). The promotive voice is focusing on the future, providing suggestions on how the organization could function more effectively, while the focus of the prohibitive voice is on the past and the present pointing out the flawed functioning. There are two common features of promotive and prohibitive voice: (1) the behavior is always voluntary, and (2) it is always driven by a helpful intention. However, leaders often feel that suggestions threaten their position (Miceli et al., 2008; Ashford et al., 2009; Morrison, 2014), and employees fear the negative consequences, such as negative performance reviews, unwanted tasks dismissal or disapprobation of coworkers (Morrison, 2011).

There are two conditions that the employees always consider when they plan the voice behavior consciously: perceived efficacy and perceived safety. The perceived efficacy is low when employees experience that their voice is not heard by the leaders. The perceived safety is high when colleagues and the leader of the team are open to opinions and critiques. Perceived leadership openness indicates how organizational culture welcomes or suppresses the subordinates’ suggestions and ideas, and it is an important predictor of employee voice behavior (Ashford et al., 1998). Psychological safety and leader’s openness are especially important because they influence the employees’ willingness to voice through their perception of safety and efficacy of voicing. Intrinsic work motivation, and affective commitment also contribute to the willingness to voice (Tangirala and Ramanujam, 2008; Liang et al., 2012; Edmondson and Lei, 2014).

Psychological safety means the extent the employee believes that their colleagues do not punish or misunderstand him or her if he or she takes a risk (Detert and Burris, 2007). When the level of psychological safety is high in a team or in the organization, we should assume that voice behavior is present (Liang et al., 2012; Edmondson and Lei, 2014). According to Lechner and Tobias Mortlock (2022) online communication difficulties (e.g., harder to explain a problem via phone or online, harder to reach coworkers, feeling of alienation) can reduce psychological safety, and voice.

Work motivation, especially intrinsic work motivation, is also an important predictor of employee voice behavior (Uğurlu and Ayas, 2016; Jaaffar and Samy, 2023). During WFH the largest threat to motivation is the lack of face-to-face contact with colleagues (Hertel et al., 2005; Bočková and Lajčin, 2021; Schade et al., 2021). However, Schade et al. (2021) and Kohont and Ignjatović (2022) found that a lot of respondents reported that they have more autonomy at work than before, which increased intrinsic motivation, during the WFH.

Finally, affective organizational commitment, which indicates a person’s emotional connection, involvement, and identification with the organization, is associated with intrinsic work motivation (Kuvaas, 2006; Galletta et al., 2011) and is a predictor of voice behavior (Nisar et al., 2020; Cheng et al., 2022). Affective organizational commitment diminishes at the home office due to psychological isolation (Wang et al., 2020; Simon et al., 2023).

### Leadership openness

Leadership openness affects voice behavior directly (Lebel, 2016; Guo, 2017) and through psychological safety (Detert and Burris, 2007), intrinsic work motivation (Siyal et al., 2021), and organizational commitment (Choi et al., 2015). As we could see, psychological safety, intrinsic motivation and affective commitment can be seriously hampered by mandatory WFH. Reintzeig (2020) mentions several traps leaders can fall in, including that they try to control every decision and centralize the answers to the crisis, and not listening to employees. Leaders should find new ways to conteract these difficulties. New, two-way communication methods are necessary with more meaningful feedback in both directions (Marino and Capone, 2021). Several authors suggest that leaders, indeed, change their leading style during crisis (e.g.: Nangia and Arora, 2021; Ajemba, 2022; Saleem et al., 2022). According to Stoker et al. (2022) managers perceived a decrease in the degree of exercising control and employees also perceived that managers significantly decreased their control during COVID 19. Balasubramanian and Fernandes (2022) identified and confirmed a crisis leadership model for which leaders’opennes proved to be an important contributor. Furthermore, Lebel (2016) suggests that external threat is positively associated with employee voice if supervisors are open to suggestions, so leadership openness is able to counteract obstacles of employees’ voice during WFH situation. He argues that when an external threat occurs employees tend to focus on the leader’s behavior and try to find guidance on which behavior is appropriate in the situation. Therefore, their supervisor’s openness to input provides an important signal that the suggestions and critiques of the employees will be considered and that these suggestions possibly lead to change.

In order to understand effective coping with the crisis, we have to take into consideration the beneficial contribution of employees as well, considering the importance of a two-way communication flow. Expectedly, if leaders show more attention to employee voices the frequency of employee voice behavior will increase.

**Figure 1:**
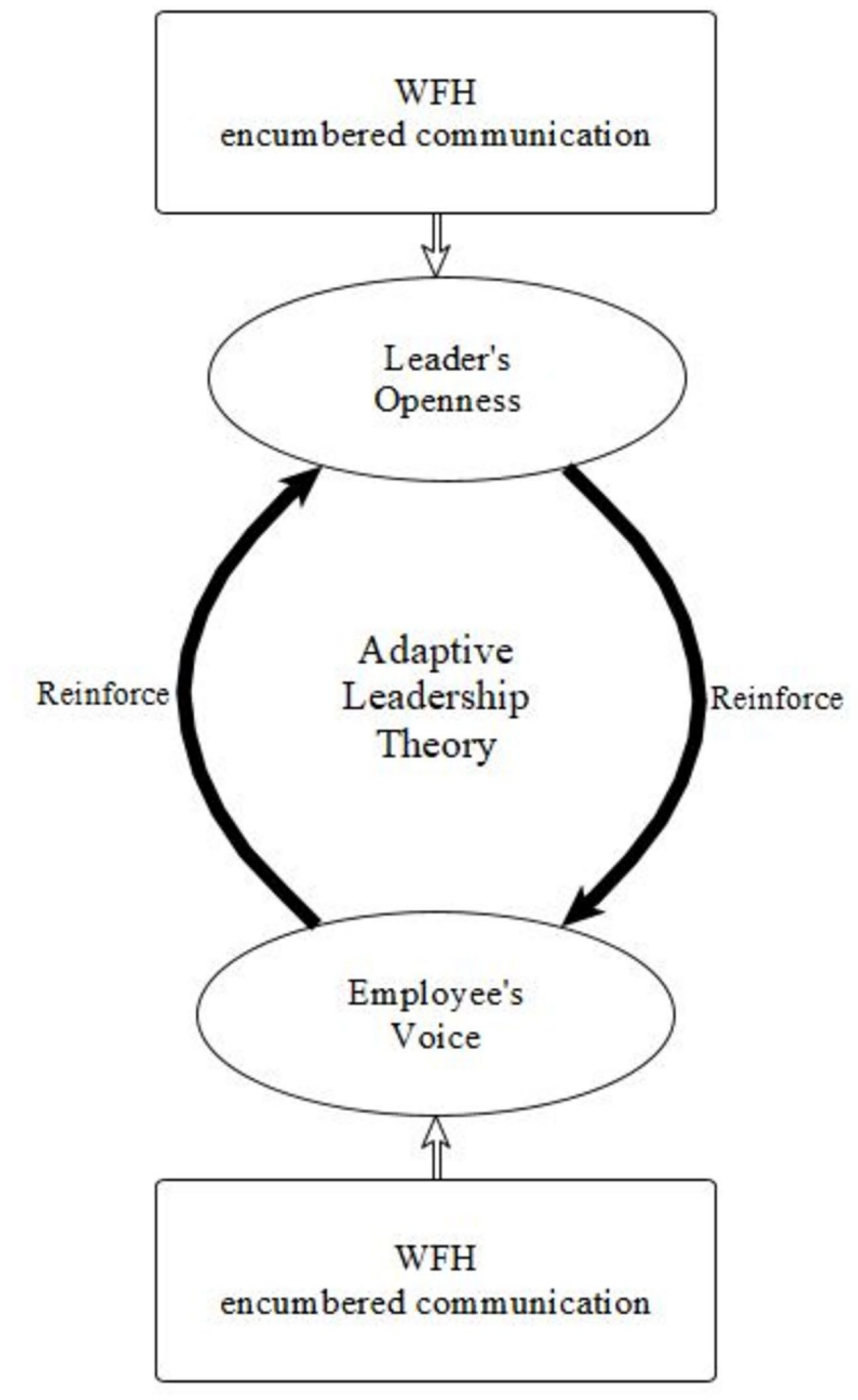
The interactive effect of leadership openness and employee voice behavior during WFH.

We propose, that in an organization when people spend more time in WFH, the leaders tend to recognize the need and the importance of being open to suggestions, and the employees will be more active and voice their suggestions and concerns. In our view, the perceived openness of the leader will be a central determining factor. A supervisor who is open to suggestions and ideas, and who experiences the beneficial effect of employee suggestions will encourage voice behavior, and voice will appear to be more effective and less risky. On the other hand, employees’ increased voice behavior will increase the openness of leaders to listen to it. Leadership openness will positively influence psychological safety, motivation and commitment, and create an environment where employees feel free to voice. We try to detect the mutual influence of employees’ voice and leaders’ openness in our study (see Figure 1).

### Hypotheses

In a crisis situation requiring adaptation, leading-following interaction changes to a more flexible leading-following process, where followers take up leadership roles and leaders take up follower roles (DeRue, 2011). According to DeRue (2011) the magnitude of the environmental change and the variability in the pattern of leader-follower relationships should be proportionate. Therefore, …

**H1**: The more time employees spend in home office, leaders will become more open to suggestions and critique because leaders need more feedback from the employees to adapt to the challenges of the crisis (Hirak et al., 2012; Balasubramanian and Fernandes, 2022).

**H2**: In WFH leaders’ openness directly increase employees voice behavior. External threat is positively associated with employee voice if supervisors are open to suggestions when external threat is present (Lebel, 2016; Stoker et al., 2022).

**H3**: Employee voice retroact and increases leader’s openness due to the reciprocal influence pattern (DeRue, 2011).

**H4**: The more employees consider their supervisor open, the more employees’ intrinsic motivation and affective commitment rise (Aubé et al., 2007).

**Figure 2:**
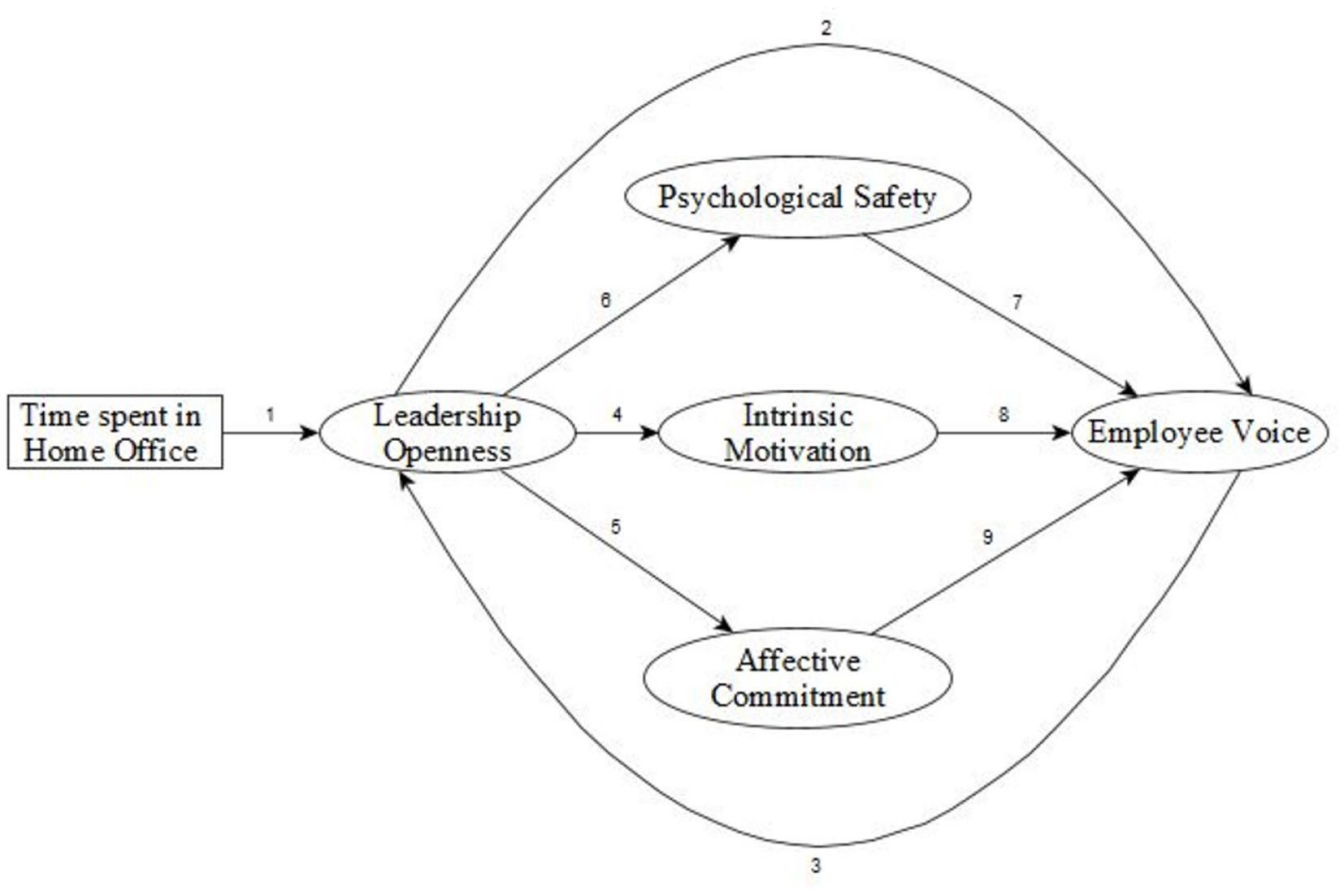
Schematic illustration of the hypothesized model. H1: 1, H2: 2, H3: 3, H4: 4 and 5, H5: 6 + 7, 4 + 8, 5 + 9.

**H5**: Psychological safety, intrinsic work motivation, and organizational commitment will moderate the relationship between leadership openness and voice behavior (Detert and Burris, 2007; Choi et al., 2015; Siyal et al., 2021). Figure 2 shows the schematic illustration of the hypothesized model.

## Materials and methods

### Participants

Employees who participated in our research corresponded to both conditions: (1) they have been a member of their current organizations for at least 2 years and (2) they spent at least a part of their work time in home office between March 2020 (the first wave of COVID-19 in Hungary) and April 2021 (time of data collection, during the third wave of COVID when rather serious restriction and WFH were still in effect in Hungary). After data cleaning, the data of 424 participants were analyzed (224 female and 200 male). 200 members worked in the public sector and 174 in the private sector. The age distribution of our data was balanced (17.9% were between the age of 18 and 30, 23.8% were between the age of 31 and 39, 28.3% were between the age of 40 and 49, 27.1% were between the age of 50 and 64 and 2.8% were older than 65). The distribution of work time spent in home office was also balanced (26.9% spent 0–20% of working time in home office, 20.0% spent 20–40%, 16.7% spent 40–60%, 14.2% spent 60–80,% and 22.2% spent 80–100%). Concerning organization size and economic sector, the research sample indicated heterogeneous distribution. A multidisciplinary market research company carried out the data collection.

### Measures

All measures not available in Hungarian were translated according to the translation guidelines by Beaton et al. (2000).

Time spent in home office was measured by the following question: What percentage of your total working time has been spent in home office since March 2020? The responses were categorized into five ordinal categories with the following values: 1–0% (the few respondents who reported 0%, were filtered out), 2 – up to 20%, 3 – up to 40%, 4 – up to 60%, 5 up to 80%, and 6 – up to 100%.

To measure employee voice behavior, we used the scale of Liang et al. (2012). Their scale consists of two subscales, promotive voice, and prohibitive voice. Both of the subscales contain five items. To shorten the scale, we abandoned one item from each of them based on the lowest factor loadings reported in the article of Liang et al. (2012). We measured the two factors on a five-point Likert scale in the questionnaire. Both subscales had a satisfactory internal consistency (Cronbach’s alpha: Promotive voice = 0.87, Prohibitive voice = 0.86).

For psychological safety, we used the items of Liang et al. (2012). In their five-item scale, they merged the items of two previous scales (Brown and Leigh, 1996; May et al., 2004). We added a new item to the original scale because we thought that emotions are missing from the safety scale (“In my workplace, I can express my true feelings),” and rephrased one item to be reverse coded (original item: In my work unit, I can freely express my thoughts., rephrased item: In my work unit, I cannot express freely my thought). We measured psychological safety on a five-point Likert scale in the questionnaire. The scale had a satisfactory internal consistency (Cronbach’s alpha: 0.70).

Leadership openness was measured through two items by Ashford et al. (1998) on a five-point Likert scale (Cronbach’s alpha: 0.91) According to Eisinga et al. (2013) the application of two-item scales can be feasible. In this case, they recommend the Spearman-Brown Coefficient to analyze reliability (Spearman-Brown Coefficient: 0.91).

Motivation was measured by the Multidimensional Work Motivation Scale (Gagné et al., 2015). The original scale was translated into Hungarian by Matuszka et al. (2018). The 19-item scale contains six factors: amotivation, material external regulation, social external regulation, introjected regulation, identified regulation, and intrinsic motivation. All factors consist of three items, except introjected regulation which consists of four. For the measure of work motivation, we used a seven-point Likert scale. In our model, we used the items of intrinsic motivation (Cronbach’s alpha: 0.89).

Organizational commitment was assessed by the classic Three-Component Model Employee Commitment Scale (Meyer and Allen, 1991, 2004). The original survey was translated into Hungarian by Kiss et al. (2012). The three factors of the scale (affective commitment, continuance commitment, and normative commitment), were measured with four items on a six-point Likert scale. The internal consistency of the three subscales were satisfactory (Cronbach’s alpha: affective commitment = 0.94, continuance commitment = 0.78, normative commitment = 0.87).

### Statistical analysis

Data was first exported to SPSS 28.0 for preliminary analysis (e.g., demographics, means, standard deviation, correlations and estimation of internal consistency). Then, measurement models were developed to discriminant- and convergent validity of the scales. The purpose of convergent validity is to test whether or not the items measure the same concept. Convergent validity consists the average variance extracted (AVE) and composite reliability (CR). These indicators are accepted when AVE > 0.5 and CR > 0.70 (Hair et al., 2017). The discriminant validity was checked using the Forner-Larcker criterion, i.e., we checked the square root of AVE. All data were collected in the same survey, therefore the findings could be attributed to common method bias. To deal with this concern, we used the common latent variable approach (Podsakoff et al., 2003).

To test the hypotheses we developed structural equation models (SEM) using Amos 24.0. *P*-values, confidence intervals, *β*-values were calculated. Acknowledging the possible non-normality of the data, the robust maximum likelihood estimator was selected. In accordance with the suggestions of Preacher and Hayes (2008) 5,000 bootstrap replication samples were applicated. The usage of bootstrapping methods is advised when the size of the is small-to-moderate (Shrout and Bolger, 2002). In evaluating the model, three goodness-of-fit indices were checked with their proper good or acceptable cut-off values (Hu and Bentler, 1999): the Comparative Fit Index (CFI; ≥0.95 good, ≥0.90 acceptable), the Tucker–Lewis index (TLI; ≥0.95 good, ≥0.90 acceptable), the root-mean-square error of approximation (RMSEA; ≤0.06 good, ≤0.08 acceptable).

## Results

Descriptive data, correlations are presented in Table 1. Table 2 indicates the results for validity and reliability based on CFA. We calculated CR, AVE and factor loadings of each investigated factor, summing by the average score to measure the level of leadership openness, psychological safety, affective commitment, intrinsic motivation, promotive and prohibitive voice. In Table 2 you can see the results of item loadings of all constructs are in acceptable range. Average variance extracted of psychological safety did not reach 0.5, but according to Fornell and Larcker (1981), it could be acceptable if the composite reliability is above 0.7. Multicollinearity was measured by variance inflation factors (VIF) and tolerance. A VIF value more than 5.0 or tolerance under 0.2 means that there is an issue with multicollinearity (Sheather, 2009). Table 2 shows that all of the VIF values are under 5.0, which indicates that there is no multicollinearity. The results of the common latent variable approach revealed that 15% of the variance was accounted to the method factor, therefore common method variance was not an issue in our study, and we were able to analyze our hypothesized model.

**Table 1:** Descriptive statistics and correlations for the hypothetic model.

Scales	*M*	*SD*	Range	α	1	2	3	4	5	6	7
1. Time spent in Home Office	3.85	1.51	2–6	-	-						
2. Leadership openness	4.75	1.47	1–7	0.91	0.11*	-					
3. Psychological Safety	3.22	0.67	1–5	0.70	0.05	0.42**	-				
4. Intrinsic Motivation	4.99	1.36	1–7	0.89	0.06	0.33**	0.28**	-			
5. Affective Commitment	4.11	1.27	1–6	0.94	0.12*	0.36**	0.38**	0.56**	-		
6. Promotive Voice	3.20	0.78	1–5	0.87	−0.06	0.25**	0.34**	0.23**	0.23**	-	
7. Prohibitive Voice	2.95	0.84	1–5	0.86	−0.07	0.15**	0.29**	0.20**	0.15**	0.63**	-
8. Gender - control	-	-	-	-	0.01	−0.00	−0.05	−0.07	−0.09	0.01	0.04

M, mean; SD, standard deviation. Gender: 1: woman, 2: man. Implied correlations are presented in the supplementary. α: Cronbach’s Alpha.

** *p* < 0.01.

* *p* < 0.05.

**Table 2:** Factor loadings, convergent validity and discriminant validity results.

Scales	Indicators	S/L	SSL	SSSL	No. I	CR	AVE/CV	Sqrt AVE/DV	VIF
1. Time spent in Home Office	T HO	1	1	1	1	-	-	-	1.1
2. Leadership openness	LO 1	0.91	0.83	1.68	2	0.96	0.84	0.92	3.6
	LO 2	0.92	0.85						3.7
3. Psychological Safety	PS 1	0.71	0.50	1.92	6	0.70	0.32	0.57	1.6
	PS 2	0.38	0.14						2.0
	PS 3	0.32	0.10						1.2
	PS 4	0.37	0.14						1.8
	PS 5	0.79	0.62						1.8
	PS 6	0.64	0.41						1.8
4. Intrinsic Motivation	INT 1	0.80	0.63	2.21	3	0.93	0.74	0.86	2.3
	INT 2	0.90	0.81						3.3
	INT 3	0.88	0.77						3.2
5. Affective Commitment	AFF 1	0.90	0.82	3.17	4	0.96	0.79	0.89	3.0
	AFF 2	0.93	0.87						4.3
	AFF 3	0.90	0.81						4.9
	AFF 4	0.82	0.67						4.4
6. Promotive Voice	PMV 1	0.83	0.69	2.56	4	0.91	0.64	0.80	2.7
	PMV 2	0.87	0.76						3.0
	PMV 3	0.82	0.66						2.5
	PMV 4	0.67	0.45						1.8
7. Prohibitive Voice	PHV 1	0.80	0.63	2.43	4	0.95	0.61	0.78	2.1
	PHV 2	0.76	0.58						2.4
	PHV 3	0.80	0.64						2.2
	PHV 4	0.76	0.58						2.5

S/L: standardized loadings, SSL: square standardized loadings, SSSL: sum of square standardized loadings, No. I.: number of indicators, CR: Composite Reliability, AVE/CV: average variance extracted, Convergent Validity, SqrtAVE/DV: square root of average variance extracted, Discriminant Validity, VIF: variance inflation factor.

**Figure 3:**
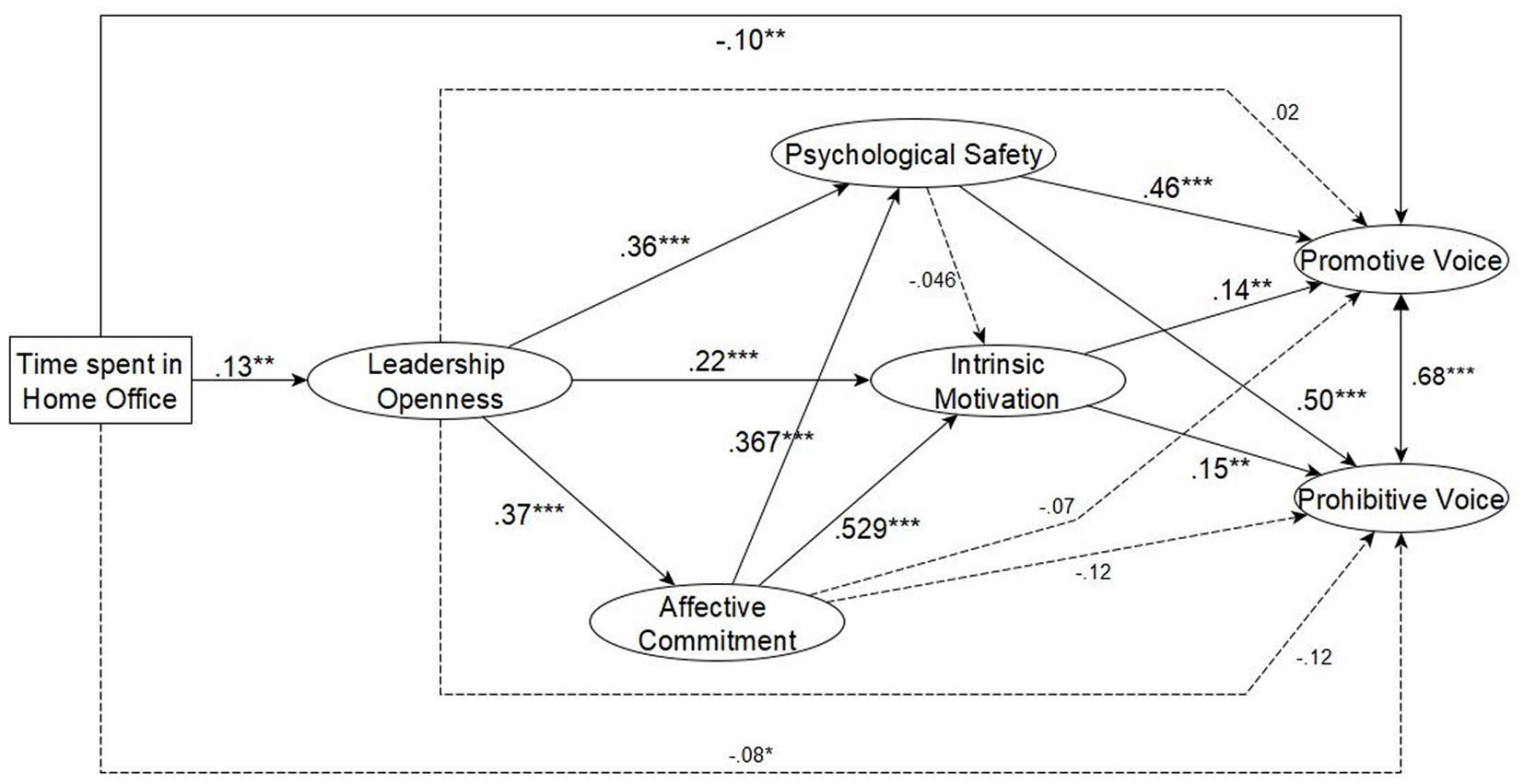
Structural equation model of the effect of time spent in home office on leadership openness, promotive- and prohibitive voice, and the mediators: psychological safety, intrinsic motivation, affective commitment. ***p < 0.01 and ** *p* < 0.05.

We created three SEM models to test the hypotheses. Our first model (M1) (see Figure 3) where we used leadership openness, psychological safety, intrinsic motivation and affective commitment as mediators between time spent in home office and voice behavior showed acceptable fit to the data (χ^2^ = 703.530, df = 249, CFI = 0.924, TLI = 0.908, RMSEA = 0.066 [90% CI: 0.060, 0.071]). In this model, we used gender as a control variable, which had no significant effect on any of the dependent variables. In order to test the third hypothesis (H3), i.e., the retroactive effect of voice behavior on leadership openness we created a new model (M2), where we measured the effect of promotive voice and prohibitive voice on leadership openness, but this model did not show an acceptable fit to the data (χ^2^ = 156.65, df = 32, CFI = 0.950, TLI = 0.930, RMSEA = 0.096 [90% CI: 0.08, 0.11]). Then, we created a model (M3) only to measure the effect of promotive voice on leadership openness. This model showed acceptable fit to the data (χ^2^ = 26.07, df = 8, CFI = 0.987, TLI = 0.976, RMSEA = 0.073 [90% CI: 0.04, 0.10]). Table 3 shows the model fit indices of the three models.

**Table 3:** Summary of the model fit indices.

Model	χ^2^	Df	*p*	CFI	TLI	RMSEA
M1	703.530	249	<0.001	0.924	0.908	0.066
M2	156.65	32	<0.001	0.950	0.930	0.096
M3	26.07	8	<0.001	0.987	0.976	0.073

M1: The model showed in Figure 3, which was used to test H1, H2, H4, H5. M2 and M3 were developed to test H3. M2 was rejected. χ^2^: chi-square, Df: degree of freedom, CFI: comparative fit index, TLI: Tucker-Lewis index, RMSEA: root’/mean square error of approximation.

### Hypotheses testing

As expected, time spent in home office positively predicted perceived leadership openness (*β* = 0.13 [95% CI: 0.03, 0.21], *p* = 0.012), but it did not predict significantly psychological safety (*β* = −0.04 [95% CI: −0.07, 0.03], *p* = 0.362), intrinsic motivation (*β* = −0.00 [95% CI: −0.07, 0.06], *p* = 0.943), affective commitment (*β* = 0.07 [95% CI: −0.02, 0.13], *p* = 0.158) and prohibitive voice: (*β* = −0.08 [95% CI: −0.10, 0.01], *p* = 0.108). Time spent in home office negatively predicted promotive voice (*β* = −0.10 [95% CI: −0.10, −0.01], *p* = 0.029).

H2 was not supported, leadership openness did not predict promotive voice (*β* = 0.02 [95% CI: −0.07, 0.09], *p* = 0.765) and prohibitive voice (*β* = −0.12 [95% CI: −0.17, 0.03], *p* = 0.064) directly. On the other hand, H3 was partly supported. According to M3, the promotive voice predicted leadership openness (*β* = 0.26 [95% CI: 0.26, 0.85], *p* < 0.001).

H4 was supported. Leadership openness predicted intrinsic motivation (*β* = 0.22 [95% CI: 0.07, 0.31], *p* < 0.001) and affective commitment (*β* = 0.37 [95% CI: 0.21, 0.42], *p* < 0.001). Moreover, leadership openness predicted psychological safety (*β* = 0.36 [95% CI: 0.11, 0.28], *p* < 0.001) as well. Finally, H5 was partly supported because affective commitment did not have significant mediation effect on promotive voice (*β* = −0.07 [95% CI: −0.14, 0.05], *p* = 0.321) and prohibitive voice (*β* = −0.12 [95% CI: −0.20, 0.04], *p* = 0.116). However, intrinsic motivation mediated the effect of leadership openness on promotive voice (β = 0.14 [95% CI: 0.00, 0.18], *p* = 0.032) and prohibitive voice (*β* = 0.15 [95% CI: −0.00, 0.21], *p* = 0.033) and psychological safety also had a mediation effect on promotive voice (*β* = 0.46 [95% CI: 0.29, 0.66], *p* < 0.001) and prohibitive voice (*β* = 0.50 [95% CI: 0.30, 0.78], *p* < 0.001). Table 4 shows a summary of the investigated hypotheses.

**Table 4:** Hypotheses summary.

Abb.	Hypotheses	*β*-value	95% CI	*p*-value	Decision
Direct effects					
H1	T HO ➔ LO	0.13	0.03, 0.21	0.012	H1 accepted
H2/1	LO ➔ PMV	0.02	−0.07, 0.09	0.765	H2/1 rejected
H2/2	LO ➔ PHV	−0.12	−0.17, 0.03	0.064	H2/2 rejected
H3/1	PMV ➔ LO	0.26	0.26, 0.85	<0.001	H3/1 accepted
H3/2	PHV ➔ LO	-	-	-	H3/2 rejected**
H4/1*	LO ➔ INT	0.22	0.07, 0.31	<0.001	H4/1 accepted
H4/2*	LO ➔ AFF	0.37	0.21, 0.42	<0.001	H4/2 accepted
H5/0	LO ➔ PS	0.36	0.11, 0.28	<0.001	H5/0 accepted
Indirect effects					
H5/1	LO ➔ PS ➔ PMV	0.46	0.29, 0.66	<0.001	H5/1 accepted
H5/2	LO ➔ PS ➔ PHV	0.50	0.30, 0.78	<0.001	H5/2 accepted
H5/3	LO ➔ INT ➔ PMV	0.14	0.00, 0.18	0.032	H5/3 accepted
H5/4	LO ➔ INT ➔ PMV	0.15	−0.00, 0.21	0.033	H5/4 accepted
H5/5	LO ➔ AFF ➔ PMV	−0.07	−0.14, 0.05	0.321	H5/5 rejected
H5/6	LO ➔ AFF ➔ PMV	−0.12	−0.20, 0.04	0.116	H5/6 rejected

THO: Time Spent in Home Office, LO: Leadership Openness, PMV: Promotive Voice Behavior, PHV: Prohibitive Voice Behavior, INT: Intrinsic Work Motivation, AFF: Affective Organizational Commitment, PS: Psychological Safety.

* H4/1 and H4/2 direct effects are parts of H5/3, H5/4, H5/5, H5/6.

** H3 was tested by M2 and M3. H3/2 was rejected due to model fit issues.

## Discussion

To understand effective coping with the crisis, it is not enough to take into account what a leader can and should do, the beneficial contribution of employees has a similar importance. We proposed that in the crisis situation leaders and employees will work together in solving the communication difficulties caused by WFH, employees by increasing their voice, their voluntary and helpful contribution, and leaders showing more openness to listen to employees’ voice.

Let us examine the reactions and the mutual dynamics of the interaction partners in the WFH crisis situation. Leaders’ openness grows with the amount of WFH: more time the members of the organization spend at home, more leaders sense the need to listening to employees’ opinion and propositions. The importance of leadership openness is reinforced in the crisis leadership model of Balasubramanian and Fernandes (2022), as well. On the other hand, against our expectation, the crisis situation in itself does not motivate employees to increase their voice (on the contrary, WFH reduces promotive voice), and the increased openness of the leaders in itself does not motivate employees to express their opinions. The mediating factors, however, show that when leaders are open to upward communication and consider employee voices thoroughly, a psychologically safe environment develops, the risk of expressing ideas or critiques decreases, and the intrinsic motivation increases. As a result, employees will articulate both their promotive and prohibitive voice. Against our expectations, affective commitment did not have a direct effect on employee voice behavior, even though it is considered to have a positive effect on OCB (Gautam et al., 2005; Bakhshi et al., 2011). However, leadership openness enhanced affective commitment, which, in turn, augmented the feeling of psychological safety and intrinsic work motivation and affected the frequency of voice behavior through these processes. We can conclude that psychological safety and intrinsic motivation exercise a most direct influence on both promotive and prohibitive voice.

In our view, our results can be best explained by DeRue (2011) adaptive leadership theory. In the home office limited communication space employees have to be more active to alleviate the ambiguity and uncertainty of the work process. However, employees get more active only if they perceive their leaders to be more open and they feel safe and motivated. For the same reason, leaders need more suggestions from the part of the employees, therefore they become more open. It seems that to alleviate the difficulties of communication in the crisis situation, leaders should initiate the shift of the usual leader-follower role behavior. However, employees voice retroacted to leader’s openness, which, in turn, can further increase employees’ inner motivation, safety and voice, so the mutually reinforcing process of leadership openness and employee voice took place. This mutual influence is in line with Stoker et al. (2022) results showing that both leaders claim that they change their leadership style to exercise less control, and both employees acknowledge that they are submitted to less control. We have to notice that only promotive voice increased leader’s openness, the critical edge of the prohibitive voice remains probably unwelcome during crisis as well.

In our research we could demonstrate the double interact perspective and take into consideration the contingent nature and the mutual influence patterns and feedback loops of leaders-employees exchange. What is new evidence in our research is that in the WFH situation the openness of the leader is growing with the amount of time spent at home and also with the amount of promotive voice manifested by the employee.

Siyal et al. (2021) argue that social exchange theory explains this effect. When employees perceive that their leader is accessible and open to their input they tend to identify with the common goal of the group and increase the effort they are willing to make to achieve these goals. As a result, employee voices can be more frequent. According to DeRue (2011) the advantage of social exchange theory is that it takes into account leaders-followers mutual relationship, but it considers leaders and followers as constant nonchanging entities. Adaptive leadership theory is based on social interactionist perspective and assumes that individuals are dynamic entities who change their leader – follower roles due to the circumstances. “As the environment becomes increasingly dynamic, a more fluid and variable pattern of leading and following should enable groups to adapt more effectively.” In DeRue’s (2011) opinion, the integration of exchange and social interactionist perspective would advance a more comprehensive theory of leadership.

In this research, we could not directly examine the openness of the leader, only through the testimony of the employees, which is considered a legitimate way to make a conclusion about a leader’s characteristics and behavior. However, there is a possibility that in the WFH situation the increase in leaders’ openness is only the perception of the subordinates. In a reduced communication space the perception of leaders’ intentions and behavior is very important, employees need to make sense of this abridged information environment and form attribution about the intentions of their leaders and colleagues. It is also a possible explanation that employees who experienced an increased possibility of decision-making and autonomy due to the diminished amount of supervision during WFH, attributed this possibility to the openness of their supervisors. Leadership openness is viewed as a sign of organizational and leader’s support, goodwill, and caring, and will positively influence psychological safety, work motivation, and work engagement (Detert and Burris, 2007; Carmeli et al., 2010; Hirak et al., 2012; Choi et al., 2015; Wang et al., 2020; Siyal et al., 2021). Even if leadership openness is only an illusion, it does not change the lesson of our result: as a practical consequence, it is worthwhile to call leaders attention to this unique possibility to motivate employees to voice their opinion and concerns. Leaders should be open to listening to these voices and by meeting expectations and being open they can make up for the lost direct feedback and regain their lost control.

### Limitations and further research

There are a number of limitations that need to be acknowledged. We are aware that a cross-sectional design is not completely adequate to study dynamic interactions, and it does not allow for causal conclusions. Although our central tenet implies a mutual influence of employees and managers over time, cross-sectional design does not allow for tracing changes over time and changing experiences of leaders and followers. We could not follow up the effect of employees’ voice on leaders’ behavior nor the effect of leaders’ growing openness on employees’ intention to voice. Moreover, as people adapt to the WFH, they can find new ways of communication and problem-solving apart from voicing. Second, the study was based on a questionnaire which could lead to distorted results because of potential biases (e.g., social desirability). Third, although we made an effort to include employees from variable fields in the research, our sample was not representative. Fourth, we could not analyze the impact of different previous WFH experiences of employees. However, before the pandemic, WFH were rather rare in Hungary, most of the people we studied had no familiarity with this arrangement. Fifth, as we have already mentioned, we could not separate employees’ perception from real changes in leaders’ openness. Finally, thought our data collection took place during the third wave of the pandemic, the perspective on what occurred in the initial weeks and months might be biased by knowing how everything has evolved since then, so hindsight bias cannot be excluded.

It is very possible that our results are culturally bound to Hungarian organizational culture characterized by large power distance and uncertainty avoidance (Bakacsi and Sarkadi-Nagy, 2003). In countries where employees’ rights and autonomy are more culturally imbedded, workers might initiate voice more actively. It would be worthwhile to study the effect of crisis on employees’ voice in a different cultural context. Despite these limitations, the findings of this research increase our understanding of the changes in voice behavior due to WFH.

It would be important to examine our research question with a longitudinal design. With a longitudinal study, we would know how behavior changes occur due to WFH and we could reveal the interactional dynamism in this change. Are leaders more open when acknowledging employees’ growing activity, or vice versa, do employees increase their voice to enhance communication? How long does this mutual beneficial process last? Second, it would be important to replicate the results in a more diverse sample. Third, including followership orientation would be an interesting development for this study. According to Carsten et al. (2022), coproduction followership orientation is an important antecedent of voice behavior, but WFH could cause a decrease in motivation among coproduction followership-oriented employees due to the distance between them and the leader. It would be interesting to examine if leadership openness could moderate this effect.

Last but not least, directly studying the changes in leaders’ attitude and behavior and the changes in employees’ voice behavior within organizations due to a changing communication space, or more generally, in a crisis environment would bring to light organizations’ healthy adaptation processes during difficult, changing times.

## Data availability statement

The datasets presented in this study can be found in online repositories. The names of the repository/repositories and accession number(s) can be found in the article/Supplementary material.

## Ethics statement

The studies involving human participants were reviewed and approved by Research Ethics Committee, Faculty of Education and Psychology, Eötvös Loránd University (ELTE PPK; Reference number: 2021/222, Date: 26.04.2021). The patients/participants provided their written informed consent to participate in this study. The questionnaires were completed online in all cases and the participants had the opportunity to quit the task at any time.

## Author contributions

BB and KF contributed to conception and design of the study. BB organized the database, performed the statistical analysis, and wrote the first draft of the manuscript. KF revised and wrote additional sections of the manuscript. All authors contributed to the article and approved the submitted version.

## Funding

This research was conducted in the framework of the “Doctoral Projects 2020/2021” syndicated research funding scholarship of Eötvös Loránd University’s Faculty of Education and Psychology.

## Conflict of interest

The authors declare that the research was conducted in the absence of any commercial or financial relationships that could be construed as a potential conflict of interest.

## Publisher’s note

All claims expressed in this article are solely those of the authors and do not necessarily represent those of their affiliated organizations, or those of the publisher, the editors and the reviewers. Any product that may be evaluated in this article, or claim that may be made by its manufacturer, is not guaranteed or endorsed by the publisher.
